# Spatially organized chemical defences shape microbial communities in honey bee colonies

**DOI:** 10.1093/ismeco/ycag247

**Published:** 2026-10-19

**Authors:** Kenya E Fernandes, Aviva Levina, Elizabeth A Frost, Dee A Carter

**Affiliations:** School of Life and Environmental Sciences, University of Sydney, Sydney, New South Wales 2006, Australia; Sydney Institute for Infectious Diseases, University of Sydney, Sydney, New South Wales 2006, Australia; School of Chemistry, University of Sydney, Sydney, New South Wales 2006, Australia; Animal Genetics and Breeding Unit, A Joint Venture of NSW Department of Primary Industries and University of New England, Armidale, New South Wales 2351, Australia; NSW Department of Primary Industries, Paterson, New South Wales 2421, Australia; School of Life and Environmental Sciences, University of Sydney, Sydney, New South Wales 2006, Australia; Sydney Institute for Infectious Diseases, University of Sydney, Sydney, New South Wales 2006, Australia

**Keywords:** honey bee, chemical ecology, social immunity, antimicrobial activity, microbiome, mycobiome, honey, propolis, host–microbe interactions

## Abstract

The success of social insects relies on dense colony living, but these conditions also favour pathogen transmission. Colonies mitigate this risk through social immunity, a coordinated system of behavioural, chemical, and immunological defences. Here, we investigate how chemical defences contribute to colony-level immunity using the European honey bee (*Apis mellifera*) as a model system. Integrating microbiome profiling, antimicrobial assays, and chemical characterization across hive components, we show that antimicrobial activity exhibits clear distance decay across compartments, closely paralleling shifts in microbial communities. This spatial pattern is most pronounced for bacterial communities and hive-associated pathogenic taxa, indicating selective suppression of harmful microbes while permitting neutral or beneficial species. Chemical analyses identify mediators including H₂O₂ in honey, flavonoids in propolis and wax, and polar aliphatics in pollen as drivers of this structured immunity. Variation in antimicrobial activity and chemical composition correlates with environmental and colony-level factors, such as apiary location, hive health, and pest exposure, highlighting the ecological sensitivity of these defences. Collectively, these results demonstrate that chemical social immunity is a coordinated, spatially organized system that shapes microbial communities and supports colony function, offering a framework to link chemical defence, microbiome dynamics, and resilience of social insects under environmental and pathogen pressures.

## Graphical abstract

**Graphical abstract:**
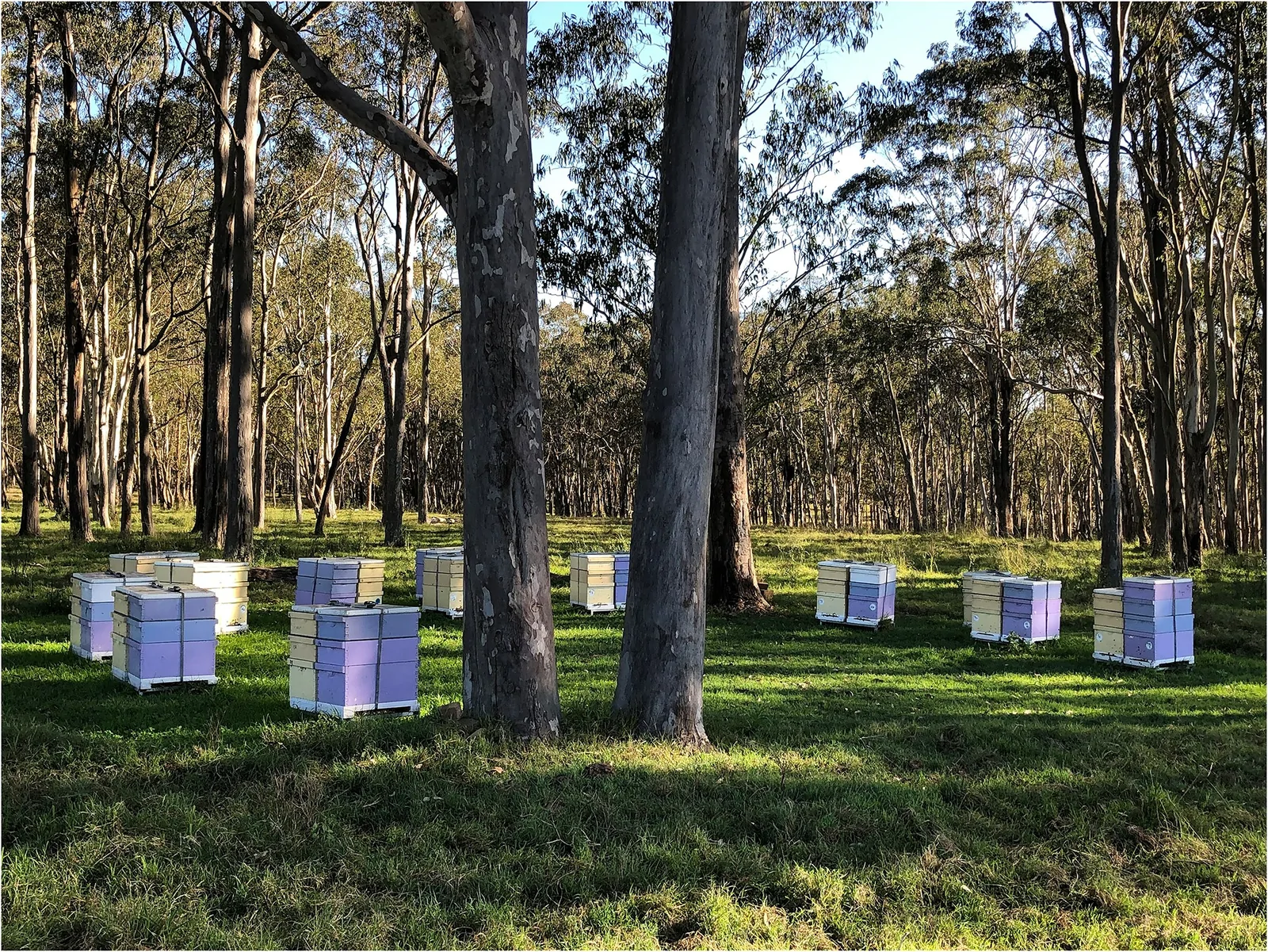

## Introduction

Social insects face a fundamental ecological challenge: the conditions that underpin the success of group living also elevate the risk of disease transmission. Frequent physical contact among nestmates, shared food resources, and stable nest microclimates create conditions that favour pathogen persistence and spread [1], placing strong selective pressure on colony-level disease control [2–4]. To mitigate this risk, eusocial species have evolved social immunity, a suite of collective behavioural, immunological, and chemical defences that reduce pathogen exposure at the colony level [5–7]. Unlike individual immune responses, social immunity arises from coordinated actions and shared resources, allowing colonies to regulate microbial communities within the nest environment rather than relying solely on host physiology [8].

Social immunity has been most extensively framed in behavioural and physiological terms, yet chemical defences constitute a central and comparatively under-integrated component of this system [2, 3]. Many eusocial insects coat nest surfaces with antimicrobial substances, either collected from the environment or produced endogenously, creating chemically hostile conditions for pathogens [9–13]. In honey bees *(Apis mellifera*), this chemical repertoire includes honey [14], propolis [13, 15], fermented pollen [16, 17], and wax [18], which are spatially arranged across brood cells, food stores, and nest substrates to form chemically distinct microenvironments. In parallel, individual bees also produce and store antimicrobial compounds in glandular secretions and body tissues that can be applied directly to nest surfaces or incorporated into hive materials [19–22]. The diversity, redundancy, and spatial arrangement of these defences suggest that antimicrobial activity is not the property of any single material but may instead arise from their coordinated deployment across the colony.

We propose that antimicrobial defences in the honey bee colony are best understood through a chemical ecology framework [23–25], in which the hive functions as a chemically heterogeneous environment structured to shape microbial communities and impede pathogen establishment. From this perspective, antimicrobial activity is not merely a biochemical trait but an ecological phenomenon that emerges from the spatial distribution of chemically distinct materials across functionally specialized nest compartments. Previous studies hint at such structuring: the hive is partitioned into distinct niches—honey storage, pollen storage, brood comb—each harbouring specific microbial assemblages, while the bee gut supports a core microbiota shaped by long-term host association [26–28]. At the colony scale, honey bees are increasingly conceptualized as holobionts, in which host, microbiota, and environment interact to influence health and fitness [29–31].

Despite this recognized complexity, most studies continue to examine antimicrobial compounds in isolation, quantifying activity without considering how chemical defences function collectively across space. Here, we test whether antimicrobial social immunity in honey bees emerges from the coordinated spatial and chemical organization of hive products and bee-derived compounds. We integrate bacterial and fungal amplicon sequencing, antimicrobial assays, and ^1^H NMR chemical profiling across multiple hive materials and bee tissues sampled from multiple colonies and apiaries. By treating hive compartments as discrete but connected microenvironments along a spatial gradient from internal bee tissues to environmentally exposed nest substrates, we assess how microbial communities, antimicrobial activity, and chemical composition covary across the colony. This integrative approach allows us to move beyond cataloguing individual antimicrobial traits and instead examines antimicrobial social immunity as a colony-level property emerging from spatial organization and functional integration among hive components.

## Materials and methods

### Sample collection

Samples were collected from 24 *A. mellifera* colonies across five apiaries at the Tocal Agricultural College Bee Research and Training Centre in Paterson, New South Wales (Supplementary Table S1) in a single sampling event. Apiaries varied in surrounding land use, ranging from managed agricultural areas to sites with greater access to diverse native flora. Hive health was assessed by professional apiary managers based on productivity, colony population, and visible pest or disease indicators; small hive beetle presence was recorded at the time of sampling, and apiary records were consulted for chalkbrood outbreaks within the preceding 6 months. From each hive, mature capped honey, propolis, fermented pollen, wax, nurse bees (from the brood area), and forager bees (from the hive entrance) were collected; bees were immediately preserved in 100% ethanol. Brood frames and hive entrances were swabbed with sterile swabs stored in Amies transport medium. Honey was stored at 4°C in the dark; all other samples were stored at −80°C. A hierarchical sampling approach was used to maximize breadth of colony representation while maintaining analytical depth across datasets. Microbiome profiling was conducted on 10 colonies and antimicrobial susceptibility testing and chemical profiling on 18 colonies, with both datasets obtained for 4 colonies.

### Amplicon sequencing and bioinformatic processing

Microbiome analysis was performed on bee gut compartments (hindgut, midgut, crop; dissected from 20 forager individuals per group after surface sterilization), fermented pollen (100 mg), and hive swabs (swab heads), all homogenized by bead beating in PBS. DNA was extracted using the DNeasy Blood & Tissue Kit (Qiagen). Paired-end amplicon libraries targeted the 16S rRNA V3–V4 region (primers 341F–805R; Illumina MiSeq v3, 2 × 300 bp; Ramaciotti Centre for Genomics, UNSW) and the fungal ITS1 region (primers ITS1F–ITS2; DNBSEQ PE300; BGI Genomics, Hong Kong), yielding *n* = 60 and *n* = 56 samples, respectively. Primers were removed using Cutadapt (v5.2) [32]; reads were quality-filtered and denoised with DADA2 (v1.38) [33] in R (v4.5.2). Full details of sample preparation, DNA extraction, and data processing are outlined in Supplementary Material S2.

### Sample extraction for antimicrobial and chemical analyses

Propolis, pollen, and wax samples (*n* = 18 for each sample type) were snap-frozen in liquid nitrogen, powdered, and extracted in 80% (v/v) ethanol at a 1:10 (w/v) ratio for 5 days at ~50 rpm (80% ethanol was selected on the basis of solvent optimization across eight solvents; Supplementary Fig. S1A). Extracts were filtered (0.22 μm), evaporated to dryness, and resuspended in DMSO at 400 mg/ml. Bee secretion extracts were recovered from the ethanol preservation medium; bee fat body extracts were prepared by homogenization of bee tissue in 80% ethanol and processed identically. Full details of sample extraction are outlined in Supplementary Material S2.

### Antimicrobial susceptibility testing by broth microdilution

Antimicrobial susceptibility testing by broth microdilution was performed in accordance with CLSI guidelines for aerobic bacteria [34], yeasts [35], and filamentous fungi [36] with minor modifications. Honeys were assayed at a concentration range of 1%–32% (w/w) diluted in either sterile water (for total activity) or freshly prepared 5600 U/ml catalase solution (for nonperoxide activity). Propolis, pollen, wax, and bee secretion extracts were assayed at a concentration range of 2–4096 μg/ml, while bee body extracts were assayed at a concentration range of 4–8192 μg/ml. Full details of test strains, culture conditions, incubation conditions, and dose–response assays are outlined in Supplementary Material S2.

### Nuclear magnetic resonance spectroscopy

Extracts in DMSO were diluted 20-fold with d^6^-DMSO (Merck cat no. 1.03424, 99.8% deuteration). ^1^H NMR spectra were collected on a Bruker Avance 500 MHz spectrometer at 300 K, using a standard ^1^H NMR setup (a sum of 64 scans), and processed by MestReNova Lite-14.0.0-23239 (MestReLab Research) and OriginPro 2022 (OriginLab) software. The spectra were calibrated and normalized using the DMSO residual peak at 2.50 ppm.

### Statistical analysis

Data were analysed using Prism 10 (GraphPad Inc.) and R (v4.5.2). Normality was assessed using the Shapiro–Wilk test. For geometric mean calculations, samples for which no MIC was reached were assigned the highest tested concentration to avoid bias in log-scale calculations. For antimicrobial correlations, MIC values were reversed so that increased antimicrobial activity produced positive correlations. Distance–decay relationships were evaluated using linear regression of Bray–Curtis dissimilarities. Pairwise correlations were assessed using Spearman’s rank correlations and visualized as hierarchically clustered heatmaps. Multivariate associations with hive metadata were assessed via PERMANOVA using the vegan package [37], with 999 permutations.

## Results

We employed a colony-level sampling design across 24 honey bee colonies spanning five apiaries within a shared geographic region (Fig. 1A). Ten colonies were sampled for microbiome analysis, including bee tissues, fermented pollen, and hive surface swabs, enabling integration of bacterial (16S) and fungal (ITS) communities across compartments within colonies. Eighteen colonies, including four overlapping with the microbiome group, were sampled for chemical and antimicrobial analysis. Chemically and functionally distinct components, including honey, propolis, fermented pollen, wax, and adult worker bees, were collected contemporaneously and processed for ethanol-based chemical extraction, antimicrobial susceptibility testing, and ^1^H NMR profiling. Hive health metrics were assessed at the time of sampling and incorporated into downstream analyses: 10 hives showed signs of low-level stress, 11 had active small hive beetle presence, and 9 had experienced chalkbrood outbreaks within the previous 6 months (Supplementary Table S1).

**Figure 1:**
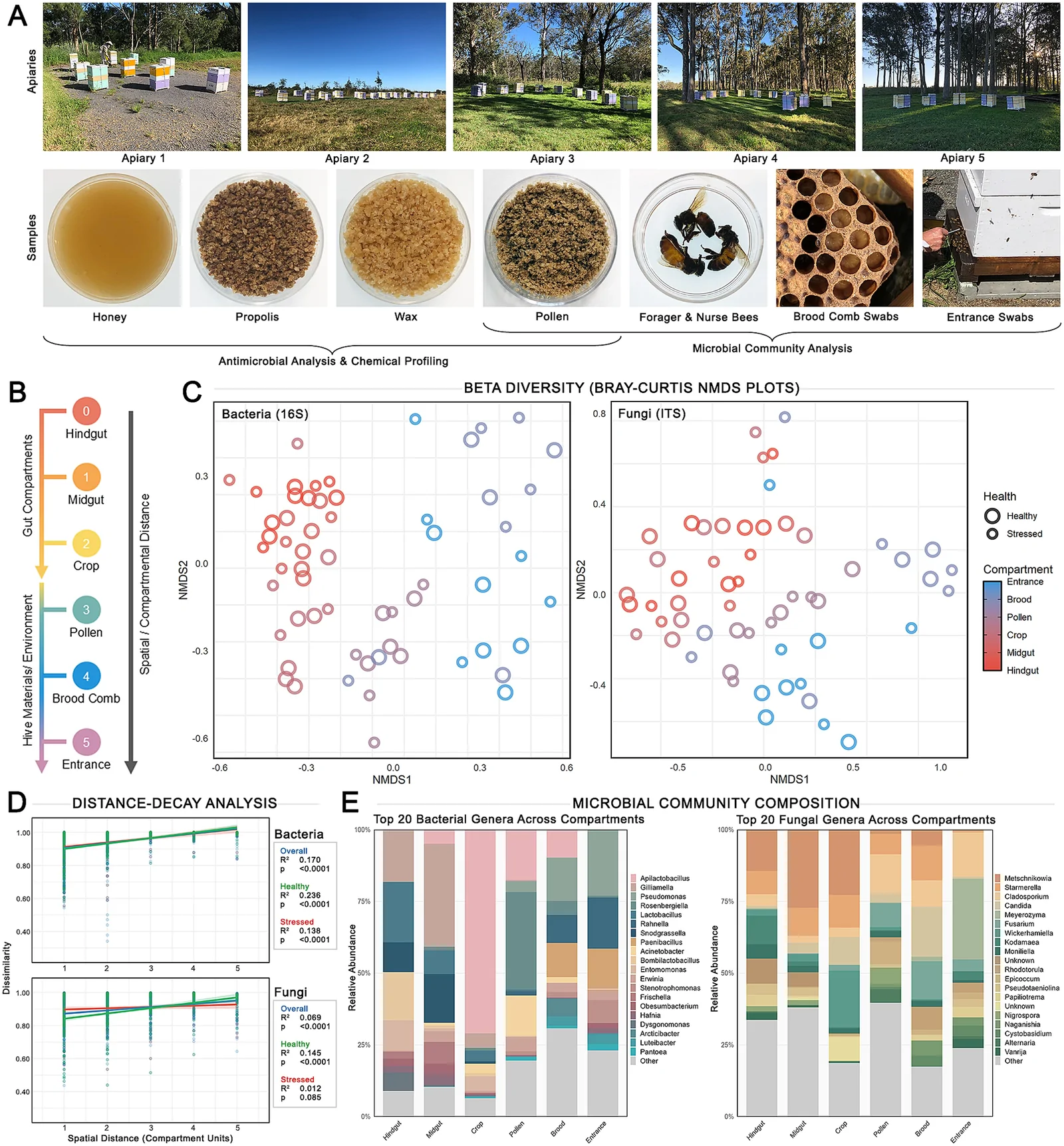
Spatial patterns and distance decay of microbial communities in honey bee hives. (A) Overview of sampling locations and sample types collected. (B) Schematic illustrating the distance metric used in analyses. Compartments were assigned ordinal values (0–5) based on their position within the hive along a continuum from internal to external. Spatial distance between any two compartments was calculated as the absolute difference between their assigned values. (C) Nonmetric multidimensional scaling (NMDS) plots using Bray–Curtis dissimilarity matrices of bacterial (left; *n* = 60) and fungal (right; *n* = 56) communities from different spatial compartments. Each point represents a sample, with a continuous colour gradient reflecting the spatial arrangement of compartments. (D) Pairwise Bray–Curtis dissimilarities between bacterial (top) and fungal (bottom) communities plotted against compartmental spatial distances. The overall trend is shown in blue, with healthy hives only in green, and stressed hives only in red. Points represent individual pairwise comparisons (*n* = 150 for bacteria; *n* = 130 for fungi). Solid lines indicate linear regressions for each group, with shading representing 95% confidence intervals. (E) Stacked bar charts showing the top 20 bacterial (left) and fungal (right) genera by relative abundance for each compartment.

### Microbial communities are spatially organized across hive compartments

To test whether microbial communities are structured along a spatially ordered continuum within the colony, we analysed bacterial (*n* = 60 samples from 10 colonies) and fungal (*n* = 56 samples) assemblages using a compartmental gradient spanning internal host-associated niches to externally exposed hive surfaces (Fig. 1B). This gradient reflects ecological and functional proximity rather than strict physical distance, capturing transitions from the bee gut through hive materials to the external environment (rationale for gradient construction in Supplementary Material S2).

Ordination using Bray–Curtis dissimilarities revealed clear spatial structuring across this gradient (Fig. 1C). Rather than discrete clusters, samples arranged progressively along NMDS axes in a manner concordant with compartment order. Gut-associated communities (hindgut, midgut, crop) transitioned through pollen and brood comb towards entrance samples, consistent with a continuous shift in community composition across the hive. This pattern was evident for both bacterial and fungal assemblages, indicating gradual turnover rather than abrupt community boundaries. Large deviations from this gradient tended to be associated with samples from stressed hives, which showed increased dispersion and displacement toward neighbouring compartments.

Distance-decay analysis supported this spatial organization (Fig. 1D). For bacterial communities, pairwise Bray–Curtis dissimilarity increased significantly with compartmental distance (*R*^2^ = 0.170, *P* < .0001). This relationship was substantially stronger in healthy colonies (*R*^2^ = 0.236, *P* < .0001) than in stressed colonies (*R*^2^ = 0.138, *P* < .0001), indicating that hive stress disrupts spatial partitioning of bacterial communities. Fungal communities showed similar but markedly weaker patterns: overall dissimilarity increased with compartmental distance (*R*^2^ = 0.069, *P* < .0001), primarily driven by healthy hives (*R*^2^ = 0.145, *P* < .0001), while stressed hives showed negligible distance decay (*R*^2^ = 0.012, *P* = .085). Alpha diversity did not increase monotonically along this gradient (linear trend: bacteria *R*^2^ = 0.024, *P* = .240; fungi *R*^2^ = 0.003, *P* = .683), indicating that the distance-decay pattern reflects genuine compositional turnover between compartments rather than a passive increase in richness along the gradient (Supplementary Fig. S2). These analyses demonstrate that both bacterial and fungal communities are spatially ordered across hive compartments in healthy colonies, with fungal taxa experiencing weaker environmental filtering.

Consistent with these spatial patterns, bacterial community composition exhibited strong compartmental structuring (Fig. 1E). Hindgut and midgut compartments were dominated by canonical honey bee-associated taxa including *Lactobacillus*, *Bombilactobacillus, Apilactobacillus, Gilliamella,* and *Snodgrassella.* The crop marked a pronounced shift, overwhelmingly dominated by *Apilactobacillus* with other gut-associated taxa declining sharply. Outside the gut, bacterial communities transitioned further toward environmentally associated and hive material–associated genera: fermented pollen and brood comb were enriched in *Rosenbergiella, Rahnella, Erwinia, Acinetobacter,* and *Pseudomonas*, while entrance samples were dominated by *Rahnella, Pseudomonas, Paenibacillus,* and other environmental taxa. Many core gut bacteria were rare or absent from these compartments, indicating strong spatial partitioning, despite likely continual inoculation of hive materials via bee activity.

Fungal communities displayed weaker compartmental differentiation and broader taxonomic overlap among hive environments. Yeasts associated with nectar- and pollen-derived substrates including *Starmerella, Metschnikowia, Wickerhamiella,* and *Meyerozyma*, were detected across multiple compartments spanning gut tissues, pollen stores, and brood-associated materials. Filamentous fungi such as *Cladosporium* and *Alternaria* were not only most abundant in fermented pollen and brood comb but also occurred at lower abundance in internal hive compartments. No fungal genus was strictly confined to a single compartment, and differences among compartments reflected shifts in relative abundance of shared taxa rather than taxonomic replacement, consistent with the attenuated spatial structuring observed in fungal beta diversity analyses.

### Diverse antimicrobial activity profiles are present across hive products and bee-derived compounds

To investigate whether coordinated antimicrobial defences across hive products, structural materials, and bee physiology suppress pathogens while preserving beneficial microbiota, we assessed antimicrobial activity against seven microorganisms: five hive pathogens (*Paenibacillus larvae*, causative agent of American foulbrood; *P. alvei* and *Enterococcus faecalis*, opportunistic secondary invaders of European foulbrood; *Ascosphaera apis*, causative agent of chalkbrood; *Aspergillus flavus*, causative agent of stonebrood) and two nonhive environmental microbes (*Staphylococcus aureus* and *Cryptococcus deuterogattii*). Six sample types representing major colony defence mechanisms were tested: honey, propolis, fermented pollen, beeswax, bee secretions, and fat bodies. While honey was assayed directly, other materials required solvent extraction for standardized assays. Eight solvents were evaluated; 80% ethanol consistently produced the strongest and most reproducible antimicrobial activity and was therefore used for all downstream analyses (Supplementary Material S2; Supplementary Fig. S1).

Minimum inhibitory concentration (MIC) analysis revealed distinct antimicrobial profiles with clear biological specificity (Fig. 2A; Supplementary Table S1). Among hive products, honey (total activity) and propolis were consistently the most potent, broadly inhibiting both bacterial and fungal pathogens. Honey (nonperoxide activity) and pollen displayed moderate activity with greater variability, while wax was the least active. Bee-derived compounds, fat bodies and secretions, were more selective, predominantly targeting hive-associated pathogens *P. alvei, P. larvae*, and *A. apis,* while largely sparing environmental microbes *S. aureus* and *C. deuterogattii.* Nurse fat bodies were generally more potent than foragers, while secretions from both castes exhibited similar activity. When organized by microbial species (Supplementary Figs S3–S5), Gram-positive bacteria associated with hive disease (*P. larvae* and *P. alvei*) were the most sensitive overall, with inhibition observed across all sample types. Hive-associated fungal pathogens *(A. apis* and *A. flavus*) showed intermediate susceptibility, with activity detected for most hive products but with greater variability and generally higher MICs. *Staphylococcus aureus* and *C. deuterogattii* were less susceptible overall, with inhibitory activity largely restricted to hive-products, and minimal activity observed for bee-derived compounds. *Enterococcus faecalis* was the most resistant species tested, inhibited only by honey and propolis.

**Figure 2:**
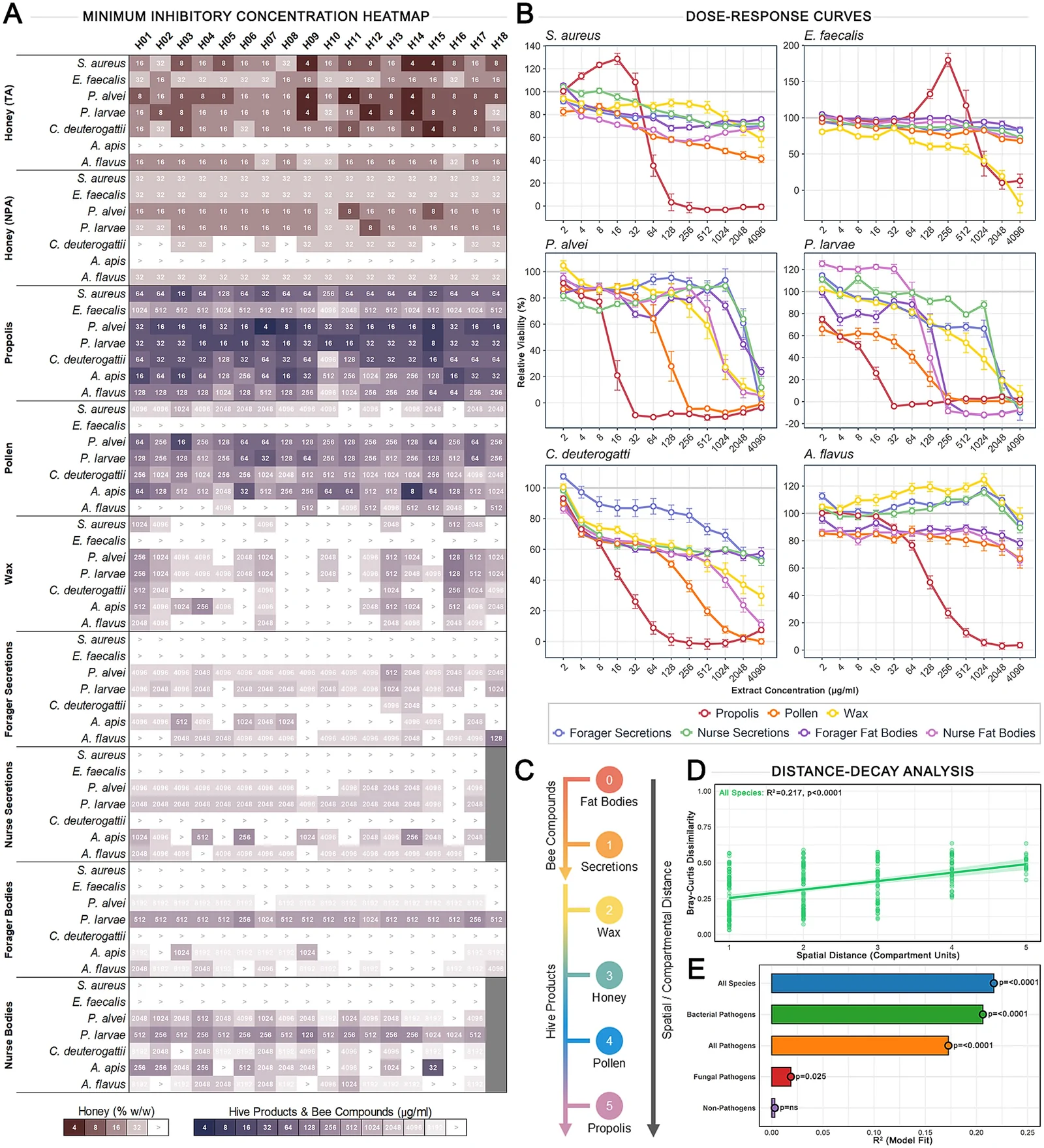
Spatial structuring of antimicrobial activity across hive compartments. (A) Minimum inhibitory concentration (MIC) heatmap for all samples, organized by sample type (*n* = 18 hives per product type; *n* = 17 for nurse bee compounds; total *n* = 143 MIC determinations). MIC values for honey samples are expressed as % (w/w) and range from 4 to 32, while MICs for hive products and bee-derived compounds are expressed as μg/ml and range from 4 to 8192. Samples with no detectable MIC within the tested concentration range are indicated as ‘>’. (B) Dose–response curves for each microbial species across seven sample types excluding honey showing relative viability (%) normalized to growth controls. Each line represents measurements averaged across samples taken from all 18 hives, with error bars showing SEM. (C) Schematic illustrating the distance metric used in analyses. Hive products were assigned ordinal values (0–5) reflecting a continuum from internal bee-derived components (center) to externally sourced resources (periphery). Spatial distance between any two compartments was calculated as the absolute difference between their assigned values. (D) Pairwise Bray–Curtis dissimilarities between all species MIC values plotted against spatial distance. Points represent individual pairwise comparisons (*n* = 270). Solid line shows a linear regression, with shading representing the 95% confidence interval. (E) Comparative analyses of distance-decay strength across microbial categories. Regression coefficients (*R*^2^) indicate the proportion of variance in Bray–Curtis dissimilarity explained by spatial distance between hive compartments.

Dose–response analyses broadly matched MIC readings but revealed additional dynamics (Fig. 2B). Propolis extracts rapidly inhibited *S. aureus* and *E. faecalis* at higher concentrations, but at certain sub-inhibitory levels, growth promotion was observed (*S. aureus* ~ 140%, *E. faecalis* ~ 200%), indicating that antimicrobial effects can be concentration dependent. Pollen extracts partially suppressed growth at low concentrations, particularly in *P. larvae*, where viability remained ~50% even at 2 μg/ml. Wax extracts at high concentrations resulted in measurable inhibition in species where MICs were not achievable: *S. aureus* viability decreased to ~70%, *E. faecalis* showed near-complete inhibition in some hive samples, and *C. deuterogattii* showed a slow, dose-dependent decrease in growth across the entire concentration range. Bee-derived compounds displayed similarly complex patterns, showing partial growth suppression (*S. aureus*, *E. faecalis*, and *C. deuterogattii*) or promotion *(P. larvae*) at sub-inhibitory concentrations.

### Antimicrobial activity exhibits distance decay along the hive compartmental gradient for pathogenic bacteria but not fungi

To investigate the spatial organization of antimicrobial activity within hives, distance-decay analyses were performed across a compartmental gradient spanning bee-derived compounds to environmentally collected materials (Fig. 2C; rationale for gradient construction in Supplementary Material S2). When all seven microbes were analysed together (Fig. 2D), antimicrobial dissimilarity increased significantly along this gradient (*R*^2^ = 0.217, *P* < .0001), indicating strong overall spatial structuring of antimicrobial activity. Stratification by hive pathogen status (Fig. 2E) revealed that this pattern was primarily driven by hive association: hive pathogens showed significant spatial differentiation (*R*^2^ = 0.173, *P* < .0001), whereas nonhive microbes showed no consistent trend (*R*^2^ = 0.003, *P* = ns). Partitioning by microbial kingdom further clarified this effect: bacterial hive pathogens exhibited much stronger spatial differentiation (*R*^2^ = 0.206, *P* = <.0001) than fungal hive pathogens (*R*^2^ = 0.0187, *P* = .025). Together, these findings demonstrate that colony antimicrobial defences are spatially tuned to target bacterial threats, while antifungal activity is more diffuse, complementing the results of the previously described MIC and dose–response patterns.

### Honey antimicrobial activity is largely driven by H_2_O_2_-mediated mechanisms

H₂O₂ concentration was measured at 2 h postdilution, a timepoint previously shown to correlate strongly with both peak and total H₂O₂ production [38]. H₂O₂ levels varied substantially among hives (5–55 ppm; Supplementary Fig. S6A) and correlated significantly with average antimicrobial activity across seven test species (Spearman’s ρ = 0.570, *P* = .014; Supplementary Fig. S6B), with higher H₂O₂ associated with lower MICs. Honey samples were applied to solid media at full strength and 50% (w/w) dilution, to allow for H_2_O_2_ production (Supplementary Fig. S6C). Samples with lower MICs generally exhibited stronger growth inhibition, and dilution to 50% w/w typically enhanced suppression. Honey was not analysed in the microbiome analysis because most resident microbes of honey are dormant rather than actively growing; however, these results are consistent with peroxide-mediated inhibition contributing functionally to microbial occupancy within the hive.

### NMR spectral profiles reveal product-specific chemical strategies

To investigate the chemical basis of product-specific antimicrobial mechanisms, we performed ^1^H NMR analysis of ethanol extracts from propolis, pollen, and wax (Supplementary Table S3). Propolis extracts displayed highly conserved spectral profiles across samples, with both high- and low-activity specimens showing consistent signals in nonpolar aliphatic (0–2.6 ppm), polar aliphatic (2.6–6 ppm), aromatic (6–9 ppm), and flavonoid hydroxyl (9–15 ppm) regions (Fig. 3A). Differences between samples were primarily quantitative rather than qualitative, reflected in signal intensity variation rather than presence or absence of major peaks. Putative major phenolic constituents were identified based on characteristic chemical shifts and coupling patterns [39, 40], including apigenin (12.96 ppm), chrysin (12.83 ppm), quercetin (12.57 ppm), kaempferol (12.49 ppm), galangin (12.36 ppm), and pinocembrin (12.13 ppm) (Fig. 3B). Pollen extracts showed broadly similar patterns across nonpolar aliphatic, polar aliphatic, aromatic, and flavonoid OH regions with differences in signal intensity (Fig. 3C). A broad peak from ~4–5.5 ppm in the polar aliphatic region was prominent in all samples, consistent with short-chain aliphatic acids or related polar metabolites [41, 42]. One notable outlier, pollen sample H04, displayed distinct propolis-like aromatic and flavonoid signals not observed in any other pollen sample, suggesting either contamination with propolis or unusual local floral resources.

**Figure 3:**
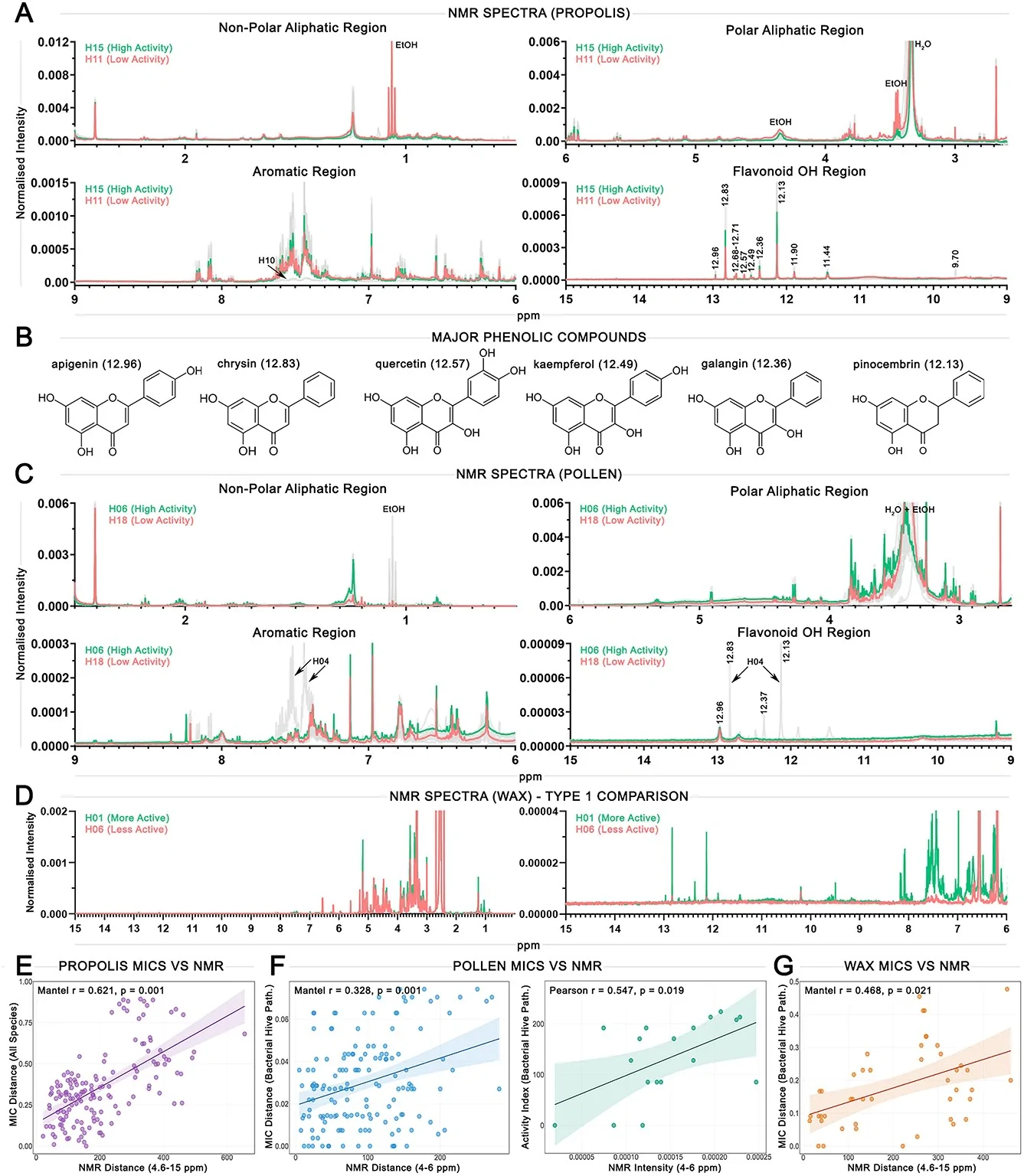
Chemical profiling of hive-derived antimicrobial materials and relationship to activity. (A) Overlay ^1^H NMR spectra of propolis extracts showing representative high-activity (H15, green) and low-activity (H11, red) samples, with annotated nonpolar aliphatic, polar aliphatic, aromatic, and flavonoid OH regions. (B) Major phenolic compounds putatively identified in propolis extracts based on chemical shifts, including apigenin, chrysin, quercetin, kaempferol, galangin, and pinocembrin. (C) Overlay ^1^H NMR spectra of pollen extracts showing representative high-activity (H06, green) and low-activity (H18, red) samples, with equivalent spectral regions highlighted. (D) Representative ^1^H NMR spectra of wax extracts illustrating variation in activity within compositional type 1, comparing a more active (H01, green) and a less active (H06, red) sample. Left panel shows full spectra, while right panel shows the magnified 6–15 ppm region. All spectra are normalized to the DMSO residue signal (2.50 ppm). (E) Propolis: pairwise NMR distance (solvent-free 4.6–15 ppm region) plotted against MIC distance between all hive pairs. Significance is tested by Mantel correlation, with each point representing a pairwise distance between two hives (*n* = 153). (F) Pollen: pairwise NMR distance (polar aliphatic 4–6 ppm region) plotted against bacterial hive pathogen MIC distance between all hive pairs (left). Significance is tested by Mantel correlation, with each point representing a pairwise distance between two hives (*n* = 153). Correlation between 4 and 6 ppm signal intensity versus activity against bacterial hive pathogens (right). Significance is tested by Pearson correlation, with each point representing an individual hive (*n* = 18). (G) Wax: pairwise NMR distance (solvent free 4.6–15 ppm region) plotted against bacterial hive pathogen MIC distance between all type 1 samples. Significance is tested by Mantel correlation, with each point representing a pairwise distance between two hives (*n* = 45). All correlation plots show linear regression fits as solid lines with shading representing 95% confidence intervals.

Wax extracts exhibited simpler and more constrained ^1^H NMR profiles compared to propolis or pollen, dominated by aliphatic signals (0–4 ppm), characteristic of long-chain fatty acids and wax esters, with less intense signals in the aromatic (6–9 ppm) and flavonoid OH (9–15 ppm) regions [41, 42] (Fig. 3D). Among samples where flavonoid signals were detectable, they were consistent with chrysin and pinocembrin, suggesting minor flavonoid incorporation into wax extracts, possibly from propolis. Extraction quality varied across samples (Supplementary Fig. S7); within the most compositionally consistent extracts, comparing a more active (H01, geomean MIC 761 μg/ml) and less active (H06, geomean MIC 3360 μg/ml) sample revealed similar polar aliphatic signals but substantially lower aromatic and flavonoid content in the latter (Fig. 3D), suggesting that flavonoids and aromatic compounds are key drivers of activity in wax, despite their lower absolute abundance.

### Antimicrobial activity is associated with distinct chemical signatures across hive products

PCA of individual propolis MIC values combined with ranked geomean MICs were used to qualitatively assign propolis samples to high-, medium-, and low-activity categories (Supplementary Fig. S8A). Corresponding PCA of NMR spectral data from the solvent-free region (4.6–15 ppm) showed overlapping distributions by activity group, with low-activity samples (H05, H10, H11, H14) occupying more peripheral positions in PC space (Supplementary Fig. S8B), indicating greater compositional divergence from the conserved propolis template. Pairwise distance analysis comparing NMR spectral distances within the solvent-free region against corresponding MIC distances revealed significant covariation between chemical composition and antimicrobial activity patterns (Mantel *r* = 0.621, *P* = .001; Fig. 3E). This indicates that propolis antimicrobial potency is associated with overall chemical composition patterns, with activity variation among hives reflecting differences in the relative balance of compounds rather than qualitative shifts in chemical class.

Pollen samples were similarly qualitatively assigned to activity categories (high, medium, or low) based on PCA of individual MICs combined with ranked geomean MICs (Supplementary Fig. S9A), but corresponding PCA of the full solvent-free NMR region (4.6–15 ppm) showed poor alignment with these activity groups (Supplementary Fig. S9B). Notably, sample H04, which displayed propolis-like aromatic and flavonoid richness inconsistent with other pollen samples, did not cluster with high activity samples providing direct evidence that aromatic compounds and flavonoids do not determine antimicrobial activity in pollen. Focusing on the polar aliphatic region (4–6 ppm) in isolation, PCA revealed strong segregation of samples by activity, most intensely for the hive pathogen *P. larvae* (Supplementary Fig. S9C). Pairwise distance analysis comparing NMR spectral distances in the polar aliphatic region against corresponding MIC distances revealed weak but highly significant correlation (Mantel *r* = 0.328, *P* = .001; Fig. 3F left) when considering bacterial hive pathogens only, but not for fungal pathogens. Linear regression analysis further showed that NMR signal intensity in this region showed stronger correlation with activity specifically against bacterial hive pathogens (Pearson *r* = 0.547, *P* = .019; Fig. 3F right). These findings indicate that pollen antibacterial activity is driven by polar aliphatic metabolites, consistent with short-chain carboxylic acids, amino acids, or related polar organic molecules [41, 42].

PCA of wax MICs showed that extracts with minimal residual solvent clustered with high and intermediate activity (Supplementary Fig. S10), indicating that extraction quality is strongly associated with antimicrobial potency. Within the highest-quality extracts, pairwise distance analysis revealed significant covariation between chemical composition and antimicrobial activity against bacterial hive pathogens (Mantel *r* = 0.468, *P* = .021; Fig. 3G). This antibacterial activity is likely driven by aliphatic and aromatic compounds based on their qualitative differing abundance between samples observed in NMR spectra, consistent with long-chain fatty acids, esters, and phenolic derivatives.

### Short- and long-chain aliphatic compounds dominate bee-derived extracts

To explore potential compositional features underlying the antimicrobial potency of bee-derived compounds, extracts from a single hive (H01) were analysed by ^1^H NMR (Supplementary Fig. S11). Secretion extracts were characterized by prominent peaks in the 4–5.5 ppm region, consistent with short-chain aliphatic alcohols and carboxylic acids [41, 42], indicating enrichment in organic acids and other low-molecular-weight compounds (Supplementary Fig. S11A). Both forager and nurse bee secretion extracts showed strong signals in this region but differed in peak number and relative intensity, suggesting possible compositional differences between castes. Fat body extracts exhibited a distinct chemical profile, dominated by intense signals in the 0.5–2.5 ppm region, characteristic of long-chain aliphatics [41, 42] while also retaining substantial signal intensity in the 4–5.5 ppm region (Supplementary Fig. S11B). This mixed profile was most pronounced in nurse bee fat bodies, where signal intensity in the 4–5.5 ppm region was markedly higher than in foragers and approached that of the aliphatic region. This may help explain the generally higher antimicrobial activity observed in nurse fat body extracts.

### Antimicrobial activity and chemical composition covary across hive products

Correlation analyses revealed strong, structured covariation in antimicrobial activity across hive products and bee-derived compounds (Fig. 4A). Antimicrobial profiles separated into two major clusters. Hive products (honey nonperoxide activity, propolis, pollen, and wax) exhibited strong positive correlations in their antimicrobial profiles across target microorganisms (Spearman *r* = 0.80–0.96), with significant correlations between wax and propolis (*P* = .049), and wax and pollen (*P* = .016). Bee-derived compounds formed a second distinct cluster, strongly intercorrelated (*r* = 0.74–0.94). Significant correlations were observed between forager secretions and fat bodies (*P* = .037), nurse secretions and fat bodies (*P* = .024), and forager secretions and nurse fat bodies (*P* = .024). Correlations between hive products and bee-derived compounds were generally positive but weaker and nonsignificant, although pollen and wax showed moderate to strong associations with nurse secretions and fat bodies (*r* = 0.67–0.85), suggesting partial functional coupling between stored materials and bee physiology.

**Figure 4:**
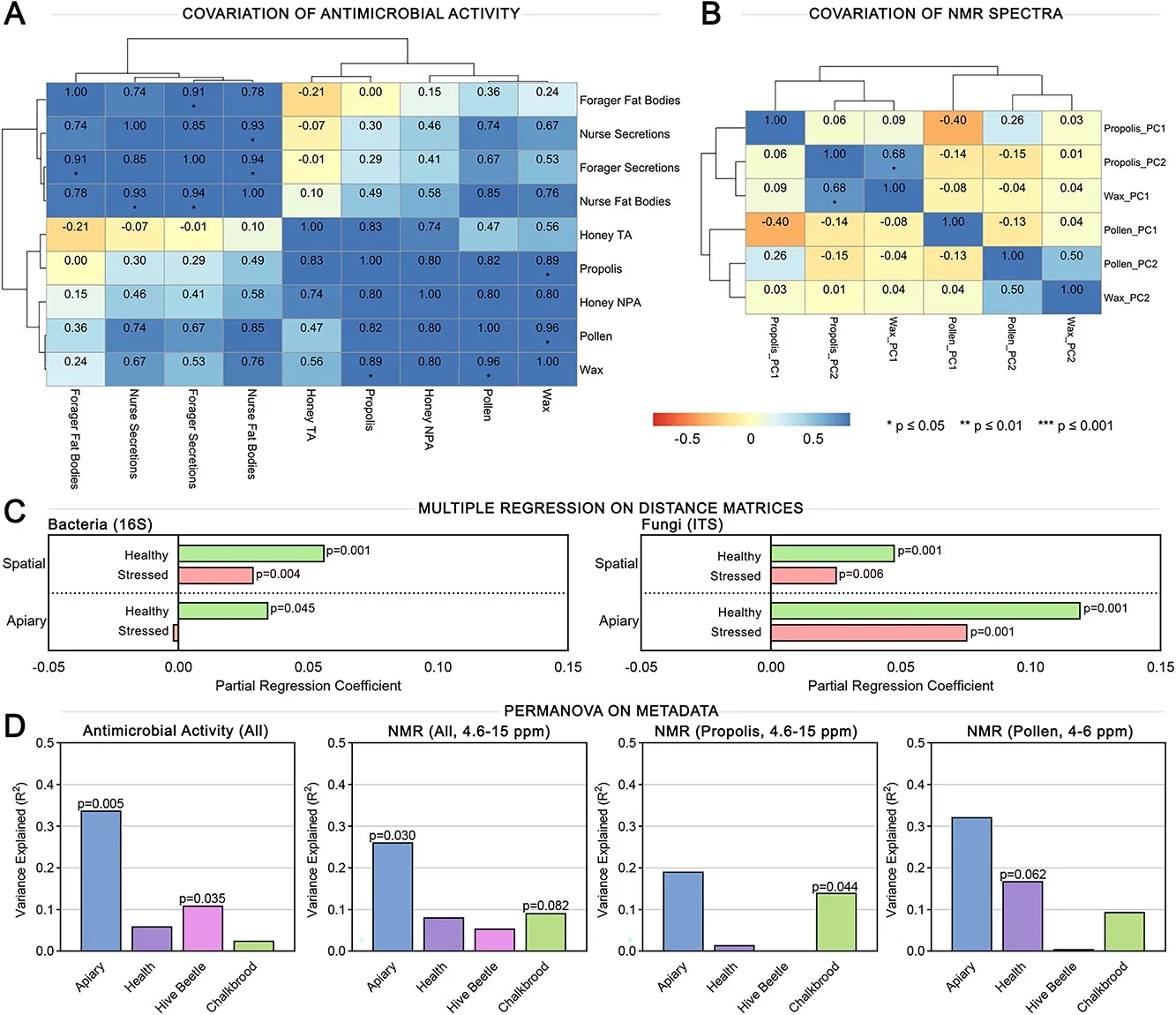
Covariation of antimicrobial activity, NMR spectra, and hive metadata. Spearman correlation heatmaps of (A) antimicrobial activity and (B) principal components of NMR spectra across sample types. Correlation coefficients are displayed with FDR-corrected significance. The colour scale represents the strength and direction of correlations. Hierarchical clustering (complete linkage) groups products with similar profiles. (C) Partial regression coefficients from multiple regression on distance matrices for microbial community dissimilarity. (D) Proportion of variance in antimicrobial activity and NMR spectra explained by each metadata factor (PERMANOVA). *P*-values are indicated for significant (*P* ≤ .05) and borderline (.05 < *P* < .10) effects. Bars represent marginal effects of each factor.

To assess covariation in chemical composition, NMR spectra were reduced by PCA such that correlations reflected coordinated chemical shifts rather than overall spectral similarity (Fig. 4B). Most PCs were largely independent; however, wax PC1 showed a moderate significant correlation with propolis PC2 (*r* = 0.68, *P* = .026) and wax PC2 a moderate but nonsignificant correlation with pollen PC2 (*r* = 0.50), indicating shared axes of chemical variation among hive products.

As our analyses established that hive health status substantially influences both bacterial and fungal community distance decay, multiple regression on distance matrices (MRM) was used to assess whether apiary identity explained additional variation in the spatial organization of microbial communities after accounting for this health-driven structuring, with analyses stratified by health group (Fig. 4C). Within healthy hives, apiary identity emerged as an additional significant predictor of 16S bacterial compositional dissimilarity (*P* = .045), reflecting local environmental structuring of the bacterial baseline, an effect that was not detectable in stressed hives. For ITS fungal communities, apiary identity exerted a strong and consistent effect regardless of health state (*P* = .001), suggesting that local environmental context shapes fungal community structure more pervasively than it does bacterial structure. Preliminary MRM analysis of antimicrobial activity indicated that hive health was not a significant driver of spatial organization, suggesting that antimicrobial profiles are not meaningfully restructured by hive stress. Given this, associations between hive metadata and antimicrobial activity or chemical composition were instead assessed directly using PERMANOVA on overall multivariate dissimilarity rather than on spatial decay structure.

PERMANOVA identified apiary as the dominant driver of variation in antimicrobial activity (Fig. 4D). Across all MIC data, apiary explained a significant proportion of variance (*R*^2^ = 0.34, *P* = .005), while small hive beetle presence accounted for a smaller but significant component (*R*^2^ = 0.11, *P* = .035). Hive health status and recent chalkbrood history were not significant predictors. A similar pattern was observed for NMR spectral data (Fig. 4D) with apiary explaining significant variation in pooled solvent-free spectra across propolis, pollen, and wax (*R*^2^ = 0.26, *P* = .030), with consistent but weaker effects at the product level. Chalkbrood history showed a borderline or significant association depending on the dataset (pooled products *R*^2^ = 0.09, *P* = .082; propolis alone *R*^2^ = 0.14, *P* = .044), while pollen chemistry showed a borderline association with hive health status (*R*^2^ = 0.17, *P* = .062). Together, these analyses indicate that broad environmental context, captured at the apiary level, is the primary determinant of coordinated variation in both antimicrobial activity and chemical composition, with disease history and hive condition contributing more modest, product-specific effects.

## Discussion

Our results show that antimicrobial social immunity in honey bees operates as a spatially organized, colony-level system rather than a collection of independent biochemical traits, providing a mechanistic framework for understanding how chemical defences may be organized across eusocial insect societies more broadly. Microbial communities, antimicrobial activity, and chemical composition vary predictably across hive compartments, forming a gradient from internal bee tissues to environmentally exposed hive surfaces. These gradients are functionally coherent: compartments facing concentrated pathogen pressure, such as bee-associated tissues, exhibit more targeted activity against bacterial hive pathogens, whereas environmentally exposed materials, such as propolis, display broader, diffuse antimicrobial profiles. The spatial organization is not static, with microbial community structure strongly influenced by hive health, and the antimicrobial activity and chemical composition of hive products shaped primarily by local environment context, reflecting the dependence of colony chemical defences on floral resources. Collectively, these findings reframe antimicrobial social immunity from a property of individual hive materials to an emergent property of their spatial integration across the colony, with defensive capacity jointly determined by colony health and environmental context.

The antimicrobial landscape is functionally stratified in line with selective immune investment theory, which predicts maximal investment against pathogens imposing the greatest fitness cost [43]. Hive-associated bacterial pathogens, particularly *P. larvae* and *P. alvei*, were consistently inhibited across hive products and bee-derived extracts, whereas nonhive-associated microbes were largely unaffected except by honey and propolis. Distance-decay analyses support this targeting: hive-associated pathogens showed significant spatial differentiation in activity (*R*^2^ = 0.173, *P* < .0001), whereas nonhive microbes showed no consistent trend (*R*^2^ = 0.003, *P* = ns). Partitioning by microbial kingdom clarified this effect further: the bacterial hive pathogens assayed exhibited much stronger spatial differentiation (*R*^2^ = 0.206, *P* < .0001) than the fungal hive pathogens (*R*^2^ = 0.0187, *P* = .025). This difference may reflect the greater threat posed by bacterial brood diseases such as American foulbrood (*P. larvae*), which represent the most devastating honey bee pathogens globally [44], compared to fungal infections like chalkbrood (*A. apis*), which typically cause sublethal or chronic impacts [45]. Broader pathogen panels would be needed to test whether this pattern holds more systematically.

Antifungal defences and fungal community structure were generally weaker and less spatially structured, consistent with evolutionary tolerance of diverse fungal communities in hives. Most hive-associated fungi are ubiquitous saprotrophs or yeasts whose persistence may be tolerated or even beneficial under normal conditions [46]. Fungi contribute multifaceted roles in honey bee colonies as pathogens, mutualists, and commensals, with pollen products hosting significant fungal communities offering protective and nutritional benefits [16, 46]. Indiscriminate antifungal investment would therefore risk disrupting these beneficial interactions, consistent with theoretical predictions that immune investment should be calibrated to net pathogen costs [47]. Apiary identity remained a strong predictor of fungal community dissimilarity regardless of health status, supporting the theory that fungi are primarily acquired from flora and environmental sources and continuously shaped by local environmental recruitment [48].

Bacterial communities showed clear spatial structuring along the compartmental gradient, with gut-associated taxa reflecting the conserved core gut microbiome of *A. mellifera* [49, 50] giving way to environmentally associated genera at hive surfaces. This spatial pattern, and its association with apiary identity, aligns with evidence that many bacteria that are present in hive materials and the crop are also found in floral nectar, indicating frequent horizontal transmission from the foraging environment [26]. The strong compartmental partitioning we observe despite this likely continuous environmental inoculation suggests active filtering rather than passive assembly. Distance decay was substantially weaker in stressed colonies (*R*^2^ = 0.138 vs 0.236 in healthy colonies), aligning with studies that show that hive stress and pathogen exposure disrupt core microbiome composition and stability [51, 52], and suggesting that spatial partitioning of microbial communities is itself a marker of colony health. That apiary identity was a significant predictor of bacterial community structure in healthy hives (*P* = .045) but not stressed hives (*P* = ns) suggests that stress-associated dysbiosis may override the locally determined environmental baseline—a loss of ecological signal analogous to that observed in other perturbed host-associated microbiomes [53–55].

The dominant driver of variation in colony chemical phenotype was apiary identity, explaining more variance in antimicrobial activity (*R*^2^ = 0.34) and chemical composition (*R*^2^ = 0.26) than hive disease or stress. This indicates that environmental context, such as local flora, land use, and geography, sets the baseline for colony chemical defence. Colonies from different apiaries consistently harboured different chemical signatures and bioactivities regardless of health, suggesting that colony-level immunity emerges partly as an ecological legacy: bees have a limited foraging range [56] and collect whatever compounds the environment provides. This aligns with studies showing strong landscape-level effects on propolis chemistry [57] and honey's antimicrobial properties [58, 59]. Chemical defences appear pre-emptive, though bees have been shown to supplement behaviourally, for example, by increasing resin collection during brood infection [13]. This, together with our previous study finding the importance of diverse forage in producing antimicrobial beehive products [58], implies that land-use intensification and monoculture agriculture may compromise antimicrobial social immunity by restricting access to the chemical diversity necessary for effective defence [60–62].

Despite product-specific mechanisms, antimicrobial profiles covaried strongly across hive materials. This suggests that there are shared upstream drivers such as dietary uptake and metabolic processing of plant-derived compounds, which have been documented for propolis-derived compounds incorporated into wax and honey [13] and for dietary phenolics in bee glandular secretions [63]. Chemical characterization illuminates the molecular basis for this coordination. Honey activity was strongly associated with hydrogen peroxide production, consistent with peroxide-mediated mechanisms as primary drivers of activity in Australian honeys [38, 58]. Propolis activity covaried with overall flavonoid-rich chemical composition, reflecting the well-established role of phenolics in propolis bioactivity [57]. Pollen antibacterial activity tracked polar aliphatic metabolites, likely short-chain organic acids, either plant-derived or generated during microbial fermentation [64, 65]. Wax antibacterial activity appears influenced by long-chain fatty acids, esters, and minor phenolic derivatives, pointing to integration of plant compounds with bee-derived metabolites. These distinct chemical strategies mean the colony deploys multiple, complementary antimicrobial mechanisms simultaneously, a redundancy that provides robustness when individual components fail.

The principles identified here—spatial stratification of chemical defences, selective targeting of ecologically relevant pathogens, and environmental-dependent variation in antimicrobial capacity—are likely to be applicable to other eusocial taxa. Ants, termites, and stingless bees also demonstrate the targeted application of antimicrobial substances to vulnerable brood, with variation in the chemistry of the nest across zones and in response to the environment [9–12, 66–68]. Whether these patterns represent convergent adaptations to shared epidemiological pressures or reflect deeper homologies in the organization of chemical social immunity remains an open question. The integrative framework developed here, combining antimicrobial assays, chemical profiling, and microbiome analysis across spatially defined nest compartments, provides a powerful new approach for addressing this question across eusocial insect societies.

Several limitations warrant acknowledgement. Antimicrobial assays employed ethanol extracts under standardized laboratory conditions, which may not replicate in-hive concentrations or interactions among materials. Sample sizes limit power for detecting smaller effects of disease variables in stratified analyses, and causal relationships between chemical composition, antimicrobial activity, and microbial community structure remain inferential.

## Conclusion

Our findings establish a systems-level framework for antimicrobial social immunity in which spatial organization, chemical diversity, and environmental context interact to produce colony-level disease resistance exceeding what any individual component could provide alone. That environmental context, captured at the apiary scale, emerges as the dominant driver of antimicrobial variation has direct implications for pollinator conservation: landscape simplification and floral impoverishment may erode social immunity not through direct toxicity but by depriving colonies of the chemical raw materials on which coordinated defence depends. These results provide a foundation for mechanistic research on the evolution of antimicrobial social immunity and for applied strategies to bolster pollinator resilience in the face of environmental change.

## Supplementary Material

Supplementary_material_ycag247

## Data Availability

Raw amplicon sequences obtained during this study are publicly available in the NCBI Sequence Read Archive under Bioproject ID PRJNA1457209. All other data that support the findings of this study are available in the supporting material of this article.
