# Supplementary material for: Spatially organized chemical defences shape microbial communities in honey bee colonies
Source: ISME Commun. 2026 Oct 19;6(1):ycag247. doi: 10.1093/ismeco/ycag247 (PMC13679945; doi:10.1093/ismeco/ycag247)
Supplement: Supplementary file 1 [file supplementary_material_ycag247.zip › Supplementary_Figures_ycag247.docx]

**Supplementary Fig S1. Solvent optimisation, reproducibility and efficiency.** (A) Samples from hive H01 were extracted using acetone (Ace), chloroform (Chl), dichloromethane (DCM), dimethyl sulfoxide (DMSO), 80% ethanol (EtOH), methanol (MeOH), mineral oil (MO), and water (H_2_O). Antimicrobial activity of each extract was assessed against *Staphylococcus aureus* and *Cryptococcus deuterogattii* to evaluate solvent-dependent effects on activity. (B) Two independent 80% EtOH extracts of each sample type were prepared from three hives (H01, H10, H17) and their antimicrobial activities against *S. aureus* and *C. deuterogattii* compared to assess reproducibility. (C) Extraction yield (%) for each sample type across all 18 hives, calculated as (mass of dried extract / mass of starting material) x 100.

**Supplementary Fig S2. Alpha diversity across the hive compartmental gradient.** Observed richness (top) and Shannon diversity (bottom) for bacterial and fungal communities across the six hive compartments. Boxplots show the median and interquartile range and points represent individual samples.

**Supplementary Fig S3. Antimicrobial activity of honey against bacterial and fungal pathogens.** Minimum inhibitory concentrations (MICs) of honey samples, including total activity and non-peroxide activity. Hive-associated pathogens are indicated with their disease name in brackets beneath species. Columns represent mean MIC values, diamonds indicate individual sample measurements (n=18), and error bars show SEM.

**Supplementary Fig S4. Antimicrobial activity of hive product extracts against bacterial and fungal pathogens.** Minimum inhibitory concentrations (MICs) of propolis, pollen, and wax samples. Hive-associated pathogens are indicated with their disease name in brackets beneath species. Columns represent mean MIC values, diamonds indicate individual sample measurements (n=18 per sample type), and error bars show SEM.

**Supplementary Fig S5. Antimicrobial activity of bee compounds against bacterial and fungal pathogens.** Minimum inhibitory concentrations (MICs) of forager and nurse secretion (top) and fat body (bottom) extracts. Hive-associated pathogens are indicated with their disease name in brackets beneath species. Columns represent mean MIC values, diamonds indicate individual sample measurements (n=18 per sample type), and error bars show SEM.

**Supplementary Fig S6. Hydrogen peroxide production and microbial profile of honeys.** (A) Violin plot showing the distribution of H_2_O_2_ concentration measured at 2 hours for honey samples and (B) correlation between H_2_O_2_ concentration measured at 2 hours and normalised MIC averaged across the 7 species. Line of best fit, Spearman’s rho, and p-value is shown. (C) Spread plates of honey either undiluted or diluted 50% (w/w) in sterile water on V8-juice or nutrient agar incubated at 35 °C for 5 days. Samples are ordered from most active starting at the top left to least active at the bottom right as determined by their average MIC.

**Supplementary Fig S7. ^1^H NMR spectra of wax extracts showing distinct compositional types.** Representative ^1^H NMR spectra of wax extracts for three compositional types: type 1 (H01, green), type 2 (H03, red), and type 3 (H18, blue). Left panels show full spectra, while right panels show the magnified 6-15 ppm region for each type.

**Supplementary Figure S8. Multivariate analyses of antimicrobial activity and chemical composition of propolis extracts.** Principal component analyses (PCA) were performed on (A) antimicrobial activity (MIC values) and (B) corresponding ^1^H NMR spectral data of the solvent-free region (4.6-15 ppm). Samples are coloured by activity class (high, medium, low) assigned based on PCA position and geomean MIC values.

**Supplementary Figure S9. Multivariate analyses of antimicrobial activity and chemical composition of pollen extracts.** Principal component analyses (PCA) were performed on (A) antimicrobial activity (MIC values) and (B) corresponding ^1^H NMR spectral data of the solvent-free region (4.6-15 ppm). Samples are coloured by activity class (high, medium, low) defined based on PCA position and geomean MIC values. (C) PCA of pollen ^1^H NMR spectral data of the polar aliphatic region (4-6 ppm) with samples coloured by activity class defined specifically by MICs against *Paenibacillus larvae*.

**Supplementary Figure S10. Multivariate analyses of antimicrobial activity and chemical composition of wax extracts.** Principal component analyses (PCA) were performed on (A) antimicrobial activity (MIC values) and (B) corresponding ^1^H NMR spectral data of the solvent-free region (4.6-15 ppm). Samples are coloured by compositional type as indicated in Supplementary Fig S7.

**Supplementary Figure S11. ^1^H NMR spectra of bee secretion and fat body extracts.** ^1^H NMR spectra of forager and nurse bee (A) secretion or (B) fat body extracts across the full spectral range (0-15 ppm) for H01. Blue boxes indicate short-chain aliphatic alcohols and carboxylic acids, and green boxes indicate long-chain aliphatics.
