# Supplementary material for: Spatially organized chemical defences shape microbial communities in honey bee colonies
Source: ISME Commun. 2026 Oct 19;6(1):ycag247. doi: 10.1093/ismeco/ycag247 (PMC13679945; doi:10.1093/ismeco/ycag247)
Supplement: Supplementary file 1 [file supplementary_material_ycag247.zip › Supplementary_Material_S2_ycag247.docx]

**Supplementary Material S2**

**Extended Results**

**Rationale for construction of the compartmental and compound gradients**

Hive compartments (Fig. 1B) and bee-derived compounds/hive products (Fig. 2C) were each assigned ordinal values (0-5) reflecting their position along a gradient from internal, host-associated material to externally-sourced or -exposed material. The two gradients were constructed independently, informed by the considerations below.

The compartmental gradient (Fig. 1B) was informed by anatomical continuity within the internal alimentary canal, the relative balance of host-derived secretions/tissues versus plant- or environmental-derived content, and the degree to which bees actively enclose or manage the material relative to the outside environment. Hindgut (0) and midgut (1) are both fully internal and anatomically continuous, harbouring the stable, host-restricted core gut microbiota, with the midgut sitting earlier in the digestive sequencing, handling more recently ingested material. Crop (2), the “honey stomach”, remains anatomically internal but functions as a storage organ for nectar immediately after ingestion, making it the innermost point where external material enters the body. Fermented pollen (3) is external in origin but modified by bee secretions during processing giving it a strong host-derived signature despite being externally stored. Brood comb (4) incorporates pollen, secretions, and other host-derived material and is continuously tended by bees, but as a fixed hive surface is exposed to more varied traffic. The hive entrance (5) is the colony’s direct boundary with the outside environment, with minimal bee filtering, and sits at the most external end of the gradient.

The compound gradient (Fig. 2C) was informed by the site of synthesis (bee tissue versus plant or environmental source), whether the material is retained on/within the bee’s body or deposited and stored externally, and, for materials of mixed origin, the extent of bee-mediated biochemical transformation of the externally-sourced component. Fat bodies (0) are bee tissue itself, the most internal category. Secretions (1) are bee-synthesised but released from the body. Wax (2) is likewise synthesised de novo by bee wax glands, but once secreted is deposited and permanently stored as external structural material. Honey, fermented pollen, and propolis (3-5) are predominantly externally-derived but differ in the degree of bee-mediated transformation before storage: honey (3) undergoes substantial bee-derived enzymatic transformation of nectar; fermented pollen (4) has bee secretions added but undergoes less direct enzymatic transformation; propolis (5) is collected plant resin with minimal further transformation, retaining much of its original plant chemistry.

**Solvent extraction with 80% ethanol produced the most consistent antimicrobial results**

While honey can be assayed directly, the remaining materials required solvent extraction to recover antimicrobial compounds for standardised assays. To identify the most effective extraction method, we compared eight solvents commonly used in the literature: acetone, chloroform, dichloromethane, dimethyl sulfoxide (DMSO), 80% ethanol, methanol, mineral oil, and water (Supplementary Fig. S1A). Solvents such as chloroform, dichloromethane, mineral oil, and water showed no detectable activity, whereas DMSO and acetone were partially effective depending on sample type. Both 80% ethanol and methanol extracts were broadly active, with ethanol consistently producing the strongest antimicrobial effect. Consequently, 80% ethanol was chosen for all subsequent analyses. Extraction reproducibility was confirmed by preparing duplicate extracts of propolis, pollen, and wax from three hives, yielding highly consistent antimicrobial activity and identical MIC values across replicates (Supplementary Fig. S1B). Final extraction yields were quantified relative to the weight of the starting material: propolis (37-58%, mean 45%), pollen (35-58%, mean 46%), wax (10-22%, mean 17%) (Supplementary Fig. S1C).

**Wax extract compositional variation is associated with extraction quality**

Three compositional types were identified based on residual solvent and water content (Supplementary Fig. S7). Type 1 extracts showed minimal water and ethanol signals, indicating effective extraction and solvent removal. Type 2 extracts contained prominent water peaks, suggesting incomplete removal, potentially due to water trapped within the lipophilic wax matrix^69^. The single type 3 extract retained ethanol signals, indicating residual solvent. PCA of wax MIC values showed that type 1 samples clustered with high and intermediate activity, type 2 with low activity, and type 3 with intermediate activity (Supplementary Fig. S10A), indicating that extraction quality is strongly associated with antimicrobial potency. PCA of NMR spectra in the solvent-free (4.6–15 ppm) region further distinguished the three compositional types along PC1, linking extraction quality directly to chemical composition (Supplementary Fig. S10B).

**Extended Methods & Materials**

**DNA extraction and sample preparation**

Microbiome analysis was performed on 60 samples from 10 colonies encompassing six types: the hindgut, midgut, and crop of bee guts, fermented pollen, and swabs from the brood comb and hive entrance. Bee guts were dissected from 20 forager individuals per group, which were surface sterilised in 1% NaOCl for 3 min and rinsed in sterile water for 30 sec. The crop, midgut, and hindgut were separated using sterile forceps and stored at –30 °C until processing. Pollen samples of 100 mg were measured out, and swab heads were removed with sterile scissors. All samples were homogenised in 500 µl of PBS with 500 mg of 2 mm glass beads in a bead beater using three cycles of 30 sec at 3000 rpm with 30 sec rests. DNA was extracted using DNeasy Blood & Tissue Kits (Qiagen) following manufacturer instructions, and concentrations were quantified with the Qubit dsDNA BR Assay Kit (Invitrogen). PCR and gel electrophoresis confirmed the presence of bacterial and fungal DNA.

**Amplicon sequencing and bioinformatic processing**

Paired-end amplicon sequencing targeted the bacterial 16S rRNA gene (V3-V4 region) and the fungal ITS1 region. 16S sequencing was performed at the Ramaciotti Centre for Genomics (University of New South Wales, Sydney) using the 341F-805R primer pair on the Illumina Miseq v3 platform (2 x 300 bp), while ITS1 sequencing was performed by BGI Genomics (Hong Kong) using the ITS1F-ITS2 primer pair on the DNBSEQ PE300 platform. Final sample numbers were as follows: n=60 for 16S samples, and n=56 for ITS samples. 16S primers were removed using Cutadapt (v5.2)^32^, and all reads were quality-filtered and denoised with DADA2 (v1.38)^33^ in R (v4.5.2). 16S reads were truncated to 250 bp (forward) and 200 bp (reverse) with a maximum of two expected errors. ITS reads were filtered with a minimum length of 50 bp and maximum of two expected errors. Denoising, paired-end merging, and chimera removal followed standard DADA2 workflows, and 16S ASVs outside 420–435 bp were discarded prior to chimera removal. Taxonomy was assigned using the BEExact database release v2023.01.30 for 16S and the UNITE database release 19.02.2025 for ITS. The 30 most abundant ASVs lacking genus-level assignments were manually queried against the NCBI nucleotide database using BLASTn, and hits with greater than 90% coverage and identity were annotated. Non-target sequences, including mitochondria, chloroplasts, and non-bacterial or non-fungal reads were removed.

**Microbiome data analysis**

ASV tables, taxonomy assignments, and metadata were imported into phyloseq (v1.54)^70^. Relative abundances were calculated within each sample and ASVs were agglomerated using taxonomic ranks from kingdom to species. Phylogenetic trees were inferred from ASV sequences using Hamming distances and neighbour-joining with the ape package^71^. Beta diversity was assessed using Bray-Curtis dissimilarities, which quantify compositional differences based on relative abundances.

**Preparation of honey samples**

Honey samples (n=18) were thoroughly mixed, incubated at 35 °C for 30 minutes to dissolve any crystals, diluted to the target concentration in sterile water, and vortexed before use. Three control honeys were included in all assays to verify assay performance: an artificial honey (ART) prepared by mixing 1.5 g sucrose, 7.5 g maltose, 40.5 g fructose, 33.5 g glucose, and 17 mL sterile water; a peroxide-based active Jarrah honey (JAR, Barnes TA 10+); and a non-peroxide-based active Manuka honey (MAN, Comvita UMF 18+).

**Preparation of propolis, pollen, and wax extracts**

Propolis, pollen, and wax samples (n=18 for each sample type) were examined for visible contaminants, and these were manually removed. Propolis and wax samples were rinsed in Milli-Q water to remove residual honey or debris, then dried with a paper towel. All samples were snap-frozen in liquid nitrogen and crushed to a fine powder. Approximately 1 g of powdered material was weighed into a 15 mL centrifuge tube, and 80% (v/v) ethanol was added at a ratio of 1:10 (w/v) of sample material to solvent. The use of 80% ethanol was selected based on solvent optimisation experiments assessing extractable antimicrobial activity across multiple solvents (Supplementary Fig. S1A). Tubes were vortexed for 30 seconds, stored in foil-lined containers to protect them from light, and placed on a shaker at ~50 rpm for 5 days. After the extraction period, samples were centrifuged to pellet solids, and the supernatant was filtered through a 0.22 µm pore syringe filter. The filtrate was transferred to pre-weighed, wide-mouth jars and left to evaporate naturally in a fume hood overnight. Extract yield was calculated by subtracting the pre-evaporation weight of the jars. Dried extracts were dissolved at a concentration of 400 mg/ml in DMSO and stored at –80 °C.

**Preparation of bee extracts**

Forager (n=18 hive samples) and nurse (n=17 hive samples) bees were preserved in 100% ethanol (approximately 1 mL per bee) and stored at –80 °C for approximately three months prior to extraction. The ethanol storage solution, containing soluble bee secretions, was processed using the same centrifugation, filtration, evaporation, resuspension, and storage steps described above. After ethanol removal, bee bodies were rinsed three times in Milli-Q water to eliminate surface ethanol. Individual bees were homogenised using a micro pestle in 500 μL of 80% (v/v) ethanol per bee, with 20 foragers and 20 nurse bees processed per hive. Homogenates were placed in a foil-lined container to protect samples from light and shaken at ~50 rpm for 5 days, then processed following the same protocol as other ethanolic extracts described above.

**Microorganisms and culture conditions**

Test microorganisms included four bacterial species (*Staphylococcus aureus* ATCC29213, *Enterococcus faecalis* ATCC29212*, Paenibacillus alvei* EMAI01*,* and *Paenibacillus larvae* EMAI02), and three fungal species (*Cryptococcus deuterogattii* R265, *Ascosphaera apis* TCI01, and *Aspergillus flavus* ATCC204304). All organisms were maintained as glycerol stocks at –80 °C. Bacterial species were grown on Nutrient Agar (NA; Oxoid) and incubated at 30 °C for 24 hours, except *P. larvae,* which was grown on MYPGP agar (120 g Mueller-Hinton broth, 15 g yeast extract, 3 g K_2_HPO_4_, 1 g sodium pyruvate, 15 g agar, 20 ml 10% glucose, 1 litre H_2_O) and incubated at 30 °C for 48 hours. Fungal species were grown on Sabouraud Dextrose Agar (SDA; 10 g peptone, 40 g glucose, 15 g agar, 1 litre H_2_O) and incubated at 30 °C for 24 hours except *A. apis* which was incubated for up to 3 weeks until good sporulation was obtained.

**Antimicrobial susceptibility testing by broth microdilution**

Antimicrobial susceptibility testing by broth microdilution in 96-well plates was performed in accordance with CLSI guidelines for aerobic bacteria^34^, yeasts^35^, and filamentous fungi^36^ with minor modifications. Briefly, inocula were prepared from colonies growing on agar plates to a final concentration of 2 x 10^5^ – 8 x 10^5^ CFU/ml for bacteria, 0.5 x 10^3^ – 2.5 x 10^3^ CFU/ml for yeasts, 0.4 x 10^4^ – 5 x 10^4^ CFU/ml for moulds. For enumeration, bacterial cultures were adjusted to an absorbance of between 0.08 – 0.1 at 625 nm using a spectrophotometer prior to dilution, while fungal cells were counted using a haemocytometer. Assays used Mueller-Hinton broth (MHB; Oxoid) supplemented with 20 mg Ca^++^/L and 10 mg Mg^++^/L for *S. aureus*, *E. faecalis*, and *P. alvei*, MYPGP broth (10 g Mueller-Hinton broth, 15 g yeast extract, 3 g K_2_HPO_4_, 1 g sodium pyruvate, 20 ml 10% glucose, 1 liter H_2_O) for *P. larvae,* yeast nitrogen base (YNB; Sigma-Aldrich) supplemented with 0.165 M MOPS and 0.5% D-glucose for *Cryptococcus*, Sabouraud Dextrose Broth (SDB; 10 g peptone, 20 g glucose, 1 litre distilled water) for *A. apis*, and RPMI-1640 (Sigma-Aldrich) supplemented with 0.165 M MOPS and 2% D-glucose for *A. flavus*.

Honeys were assayed at a concentration range of 1-32% (w/w) diluted in either sterile water (for total activity) or freshly prepared 5600 U/ml catalase solution (for non-peroxide activity). Propolis, pollen, wax, and bee secretion extracts were assayed at a concentration range of 2-4096 µg/ml, while bee body extracts were assayed at a concentration range of 4-8192 µg/ml. The final DMSO concentration in all assay wells did not exceed 1%. Positive drug controls included amphotericin B for fungal strains and tetracycline for bacterial strains. Plates were incubated without agitation at 35 °C for 18 hours (*S. aureus, E. faecalis*), 24 hours (*P. alvei, A. flavus*), 48 hours (*C. deuterogattii*), 72 hours (*P. larvae*) or 96 hours (*A. apis*). The MIC was defined as the lowest concentration of sample that inhibited visible growth. All assays were conducted with at least three independent biological replicates per sample.

**Dose-response curves**

Plates were prepared and incubated as described above, with resazurin dye (20 μl of 0.01% solution) added to each well at the end of the incubation period, followed by an additional incubation period of 1-4 hours. Absorbance was measured at 573 and 605 nm, and resazurin reduction was calculated as the difference between these wavelengths. Relative microbial viability (%) was normalised to the growth control (maximum signal) and media-only control (minimum signal) using the formula: (value – minimum) / (maximum – minimum) × 100. Dose-response curves were generated from these normalised values. Dose–response analyses were performed once per sample, with SEM calculated across independent samples from 18 hives within each sample type.

**Hydrogen peroxide (H_2_O_2_) quantification**

H_2_O_2_ production was measured in honey samples using peroxide test strips as described in ^32^. Briefly, honey samples were diluted to 25% (w/w) in sterile water and incubated at room temperature for 2 hours. Test strips (Precision Laboratories IS124-50S) were then immersed for 1 second, allowed to develop for 10 seconds, and scanned. Images were converted to grayscale in ImageJ (v1.52a) and mean gray values (0-255) were quantified. H_2_O_2_ concentrations in parts per million (ppm) were calculated using a standard curve generated from known H_2_O_2_ concentrations.

**Honey microbial content characterisation**

To assess the culturable microbial content of honey, samples were tested either undiluted or diluted 50% (w/w) in sterile Milli-Q water. A volume of 100 µl was applied to NA or V8-juice agar (Oxoid) using a truncated P1000 pipette tip, then evenly spread across the entire surface of the plate using a sterile cotton swab. Plates were incubated at 35 °C for 5 days and imaged using a digital camera. Two independent replicates were performed on different days.

**Nuclear magnetic resonance spectroscopy**

Extracts in DMSO were diluted 20-fold with d^6^-DMSO (Merck cat no. 1.03424, 99.8% deuteration). ^1^H NMR spectra were collected on a Bruker Avance 500 MHz spectrometer at 300 K, using a standard ^1^H NMR setup (a sum of 64 scans), and processed by MestReNova Lite-14.0.0-23239 (MestReLab Research) and OriginPro 2022 (OriginLab) software. The spectra were calibrated and normalised using the DMSO residual peak at 2.50 ppm.

**Statistical analysis**

The relationship between H_2_O_2_ concentration and antimicrobial activity was assessed using Spearman’s rank correlation, with linear regression used to visualise trends. For principal component analyses (PCA), MIC values were log-transformed, mean-centred, and scaled, while NMR spectra were filtered to the region of interest and Pareto-scaled prior to PCA.
